# Learning curve of distal transradial access for coronary intervention: a single-center retrospective study

**DOI:** 10.3389/fcvm.2026.1733493

**Published:** 2026-05-12

**Authors:** Feixiang Li, Huajia Yang, Liaohang Xu, Junhui Yang, Xuandong Jiang

**Affiliations:** 1 Department of Cardiology, Affiliated Dongyang Hospital of Wenzhou Medical University, Dongyang, Zhejiang, China; 2 Intensive Care Unit, Affiliated Dongyang Hospital of Wenzhou Medical University, Dongyang, Zhejiang, China

**Keywords:** coronary intervention, distal transradial access, LASSO regression, learning curve, puncture success factors

## Abstract

**Background:**

The vascular access site for coronary intervention has evolved from the femoral artery to the conventional radial artery; more recently, the distal transradial access (dTRA) approach has been established. Although dTRA is preferred owing to its association with improved patient comfort and reduced complications, it is associated with a steep learning curve. We aimed to analyze the learning curve of the dTRA approach and explore the factors associated with procedural success to gain insights shortening the learning curve and enhancing safety and efficiency.

**Methods:**

This was a single-center, retrospective observational study of 150 consecutive coronary procedures attempted via the dTRA by an experienced radial operator between April 23, 2025, and August 23, 2025. To delineate the learning curve, we analyzed the operator's success rate, number of puncture attempts, and procedure time across case intervals. The least absolute shrinkage and selection operator (LASSO) logistic regression model was used to identify the predictors of access failure.

**Results:**

The overall success rate was 87.3% (131 of 150 procedures). The spline-based learning curve analysis demonstrated a two-phase maturation process: the operator's predicted probability of success plateaued after approximately 75 cases, while measures of procedural efficiency (puncture attempts and time) continued to optimize and stabilized at approximately 100 cases. LASSO regression analysis revealed the anterior-wall puncture technique as a significant technical factor associated with success. Conversely, high baseline brain natriuretic peptide (BNP) and systolic blood pressure (SBP) levels were identified as patient-related clinical risk markers predicting procedural failure. Notably, 100 pg/mL and 10 mmHg increases in BNP and SBP levels were associated with 1.9% and 0.8% decreases in the odds of success, respectively (odds ratio = 0.98 per 100 pg/mL and 0.992 per 10 mmHg, respectively).

**Conclusion:**

For an experienced operator in radial access, proficiency in the dTRA technique was achieved after approximately 75 procedures. From a technical standpoint, promoting the anterior-wall puncture technique may effectively increase success rates. Considering the patient's perspective, high pre-operative BNP and SBP levels serve as potential risk markers predicting puncture difficulty and demand substantial attention during pre-procedural assessment. However, the validity of these findings warrants further investigation.

## 1 Introduction

Percutaneous coronary intervention (PCI) is the cornerstone of coronary artery disease treatment, and the vascular access choice directly affects procedural successes and patient outcomes (1). The shift from traditional transfemoral access to transradial access (TRA) represents a major advancement in interventional cardiology, markedly reducing bleeding complications and improving patient comfort (2). Distal transradial access (dTRA) has emerged as an innovative technique to further optimize patient benefits (3). The dTRA approach targets the distal branches of the radial artery in an anatomical snuffbox. dTRA theoretically preserves the proximal radial artery via collateral circulation, minimizing the incidence of radial artery occlusion (RAO) – the most common complication of conventional TRA – while improving hemostasis and post-procedural patient experience (4–6). These benefits have been demonstrated in randomized controlled trials and studies on complex lesions (7, 8).

Despite the advantages of dTRA, its widespread clinical adoption remains limited (9). The distal radial artery has a small diameter and marked anatomical variations, making puncture significantly more challenging in dTRA than in conventional TRA (10). This results in a relatively steep learning curve for operators (11). To date, most studies on dTRA have focused on its feasibility and safety, with insufficient quantitative analysis of its learning curve, particularly regarding the factors that influence learning efficiency and puncture success. Addressing these gaps may substantially guide trainees and help experienced operators identify high-risk patients, optimizing surgical strategies and ensuring patient safety.

Therefore, we aimed to systematically delineate the learning curve by retrospectively analyzing the first 150 consecutive dTRA procedures performed by an experienced radial operator. Additionally, using the least absolute shrinkage and selection operator (LASSO) regression model, we sought to explore the pre-procedural predictors of access failure, a novel approach for cases with minimal failure events. Our findings may provide data-driven support for standardized dTRA training programs and offer evidence-based guidance for improving safety and efficient application of this technique in clinical practice.

## 2 Methods

### 2.1 Study design and ethics

This was a single-center, single-arm, retrospective, observational study. The study protocol was approved by the Institutional Review Board of Dongyang People's Hospital (DRY-2025-YX-257). The need for obtaining informed consent was waived by the Ethical Committee of Dongyang People's Hospital because of the retrospective nature of this study, which involved no human tissue collection or storage. All procedures were conducted per the ethical principles of the Declaration of Helsinki. This report follows the Strengthening the Reporting of Observational Studies in Epidemiology statement (Supplementary Table S1).

### 2.2 Study population

We consecutively enrolled patients who underwent coronary angiography (CAG) or PCI via the attempted dTRA approach between April 23, 2025, and August 23, 2025, at the Department of Cardiology, Dongyang People's Hospital. All procedures were performed by a single experienced radial operator who had performed >5,000 independent conventional TRA procedures. Regarding prior dTRA experience, before the 150 cases included in this analysis, the operator had attempted only two dTRA procedures, both of which were unsuccessful. Following these initial failures, the operator engaged in self-directed online learning of distal radial anatomy and observed a single live dTRA procedure in the catheterization laboratory. No formal structured training, simulation, or proctorship was undertaken by the operator prior to the study period.

The inclusion criteria were as follows: age ≥18 years, clinical diagnosis of suspected or confirmed coronary artery disease with an indication for CAG or PCI, and a clearly palpable radial artery pulse in or near the anatomical snuffbox.

The exclusion criteria were as follows: known severe tortuosity, stenosis, or the occlusion of the radial artery or its distal branches; end-stage renal disease requiring hemodialysis; or a Type D result on the Barbeau Test (12).

#### 2.2.1 dTRA procedure

All procedures were performed under local anesthesia (2% lidocaine). The operator used a 20-gauge breakaway needle for puncture, applying a through-and-through or anterior-wall technique. Upon successful puncture with pulsatile blood flow, a 0.025-inch straight-tip or 0.018-inch soft-tip guidewire was inserted. Subsequently, a 6-Fr radial artery sheath (Terumo, Tokyo, Japan) was advanced over the wire. After sheath insertion, all patients received an intra-arterial cocktail solution containing 0.1 mg of nitroglycerin and 4,000 units of unfractionated heparin to prevent vasospasm and thrombosis. Post-procedure, hemostasis was achieved by applying a sterile gauze pad over the puncture site, which was secured with an elastic bandage, with the pressure being gradually released over 2–4 h.

#### 2.2.2 Data collection and outcomes

Data were retrospectively collected by reviewing the hospital's electronic medical records and picture archiving and communication systems. We collected information on patient demographics, clinical comorbidities, laboratory results, and detailed procedural data.

The primary outcome was the learning curve for the dTRA approach, assessed using the following three parameters: dTRA success rate per 25 consecutive cases, mean number of puncture attempts per 25 consecutive cases, and median procedure time per 25 consecutive cases.

The secondary outcomes included pre-procedural predictors of dTRA failure and in-hospital access-site complications, such as severe hematoma, radial artery occlusion, pseudoaneurysm, arteriovenous fistula, and nerve injury.

Regarding safety outcomes and radial artery patency, clinical follow-up was systematically conducted. Patency was evaluated via manual palpation before hospital discharge (typically 48 h post-procedure), as well as during scheduled outpatient clinic visits at 1, 3, and 6 months. For patients requiring re-evaluation, radial patency was further verified during their 12-month follow-up angiography.

#### 2.2.3 Variable definitions

Successful dTRA was defined as the placement of a guidewire and sheath into the distal radial artery without crossover to another access site. Any crossover to a conventional ipsilateral or contralateral radial artery or femoral artery was defined as a failure. While procedural success was evaluated as a binary outcome, the spectrum of procedural difficulty was quantitatively captured and analyzed using two continuous metrics: total puncture time and the total number of puncture attempts. The procedure time was defined as the duration from the injection of the local anesthetic to the complete insertion of the sheath into the vessel.

#### 2.2.4 Statistical analysis

All statistical analyses were performed using R software (version 4.2.0; https://mirrors.tuna.tsinghua.edu.cn/CRAN/). Missing values for variables at a rate of <20% were addressed using multiple imputations. Outliers were detected using the interquartile range (IQR) and treated as missing values. Continuous variables are presented as mean ± standard deviation or median (IQR) and were compared using an independent samples t-test or Mann–Whitney U test. Categorical variables are presented as frequencies (percentages) and were compared using the *χ*^2^ test or Fisher's exact test.

To identify the predictors of dTRA failure, we performed a univariate logistic regression analysis. Subsequently, all variables were included in a multivariate analysis using a LASSO logistic regression model (13, 14). Given the minimal failure events (*n* = 19) in this study, the traditional multivariate regression model was at risk of overfitting. Through L1 regularization, LASSO regression effectively selects a subset of the most valuable predictors from a large candidate pool. Internal validation was performed using 10-fold cross-validation to calculate the cross-validation error and determine the optimal penalty parameter (lambda, *λ*). In this study, we selected predictors based on the *λ*. min criterion (the *λ* that minimizes the cross-validation error) rather than the more conservative *λ*. 1se rule. This decision was made because the extreme scarcity of failure events caused *λ*. 1se to excessively shrink coefficients, resulting in a null model (underfitting). *λ*. min was therefore utilized to retain meaningful clinical signals and maximize predictive capability. Finally, the discriminative ability of the selected model was assessed by calculating the C-statistic (Area Under the Curve, AUC). The analysis was performed using the glmnet package, which implements a coordinate descent algorithm. All statistical tests were two-sided, and a *P*-value < 0.05 was considered statistically significant. Furthermore, to address the potential arbitrariness of categorical case grouping, we employed restricted cubic spline (RCS) modeling with 3 degrees of freedom to continuously evaluate the learning curve. This allowed for an objective, non-linear assessment of how procedural outcomes (success probability, attempts, and time) evolved along with the accumulating case sequence (from 1 to 150).

## 3 Results

### 3.1 Baseline characteristics and overall success rate

The study included 150 consecutive patients who underwent attempted dTRA. The cohort comprised 94 males (62.7%) with a mean age of 67.1 ± 10.5 years. The overall success rate of the dTRA approach was 87.3% (131 of 150 procedures). The 19 cases (12.7%) of dTRA failure were successfully converted to the ipsilateral conventional radial approach, and all scheduled procedures were completed without crossover to femoral access.

Patients were divided into two groups based on dTRA success or failure. The detailed baseline clinical characteristics are presented in Table 1. Univariate analysis showed no significant differences between the two groups for most baseline characteristics, including age, sex, or the incidence of hypertension or diabetes. However, there was a trend towards a higher prevalence of a history of cerebral infarction (26.3% vs. 9.9%, *P* = 0.055) and peripheral artery disease (31.6% vs. 14.5%, *P* = 0.093) in the failure group than in the success group. Additionally, the admission systolic blood pressure (SBP) was higher in the failure group than in the success group (151.9 ± 20.3 vs. 142.1 ± 18.3 mmHg, *P* = 0.060), suggesting a potential association between these factors and puncture failure.

**Table 1:** Baseline patient characteristics.

Variables	Failure (*n* = 19)	Success (*n* = 131)	*P*-value
Age (years), mean ± SD	66.9 ± 9.1	67.1 ± 10.7	0.914
Female, n (%)	6 (31.6)	50 (38.2)	0.763
Smoking, n (%)	9 (47.4)	52 (39.7)	0.699
Drinking, n (%)	6 (31.6)	40 (30.5)	1
Height (cm), mean ± SD	160.5 ± 7.2	161.9 ± 11.6	0.459
Weight (kg), mean ± SD	60 ± 12.4	65.4 ± 17.4	0.106
Body mass index (kg/m^2^), mean ± SD	23.1 ± 3.7	24.1 ± 4.2	0.264
Hypertension, n (%)	15 (78.9)	86 (65.6)	0.372
Diabetes mellitus, n (%)	4 (21.1)	41 (31.3)	0.52
Atrial fibrillation, n (%)	3 (15.8)	8 (6.1)	0.147
Acute coronary syndrome, n (%)	5 (26.3)	27 (20.6)	0.557
Cerebral infarction, n (%)	5 (26.3)	13 (9.9)	0.055
Peripheral artery disease, n (%)	6 (31.6)	19 (14.5)	0.093
Coronary artery calcification, n (%)			0.198
Grade 0	2 (10.5)	37 (28.2)	
Grade 1	4 (21.1)	37 (28.2)	
Grade 2	4 (21.1)	17 (13)	
Grade 3	9 (47.4)	40 (30.5)	
Surgery, n (%)			<0.001
CAG (Coronary angiography)	12 (63.2)	2 (1.5)	
GAG (Guidewire-Assisted Angioplasty)	2 (10.5)	79 (60.3)	
IVUS (Intravascular ultrasound)	0 (0)	2 (1.5)	
PCI (Percutaneous coronary intervention)	3 (15.8)	35 (26.7)	
PTCA (Percutaneous transluminal coronary angioplasty)	2 (10.5)	13 (9.9)	
PCI duration (min), Median (Q1, Q3)	18.7 (15.9, 32.6)	10.4 (8.3, 13.2)	<0.001
Total puncture attempts, mean ± SD	4.2 ± 1.3	1.5 ± 0.7	<0.001
Puncture time (s), median (Q1, Q3)	950 (666, 1784.5)	134 (83.5, 263.5)	<0.001
Access side, n (%)	0 (0)	4 (3.1)	1
First attempt success, n (%)	1 (5.3)	82 (62.6)	<0.001
Anterior wall approach, n (%)	0 (0)	58 (44.3)	<0.001
Sion wire used, n (%)	0 (0)	9 (6.9)	0.604
Contrast volume (mL), median (Q1, Q3)	25 (15, 25)	25 (25, 60)	<0.001
Radiation dose (dap), median (Q1, Q3)	256 (55.7, 580.1)	212.8 (95.2, 333.1)	0.982
Left ventricle end diastolic diameter (mm), mean ± SD	47.8 ± 6.3	47.6 ± 7.7	0.864
Left ventricle ejection fraction (%), median (Q1, Q3)	63 (58.5, 67)	66 (61, 70)	0.108
Brain natriuretic peptide (pg/mL), median (Q1, Q3)	127.8 (37.5, 686.6)	68.1 (29.9, 209.7)	0.2
Hemoglobin (g/L), mean ± SD	132.5 ± 18.8	132.6 ± 17.8	0.976
Platelet count(10^9^/L), mean ± SD	205.2 ± 59.1	204.9 ± 50.7	0.988
Serum creatinine (*μ*mol/L), mean ± SD	85.5 ± 33.3	72 ± 23.9	0.102
Uric acid (μmol/L), mean ± SD	346.8 ± 119.8	309.6 ± 94.2	0.209
High-density lipoprotein (mmol/L), mean ± SD	1.1 ± 0.4	1.2 ± 1.6	0.515
Low-density lipoprotein (mmol/L), mean ± SD	2 ± 0.5	1.9 ± 0.7	0.957
Alanine transaminase (U/L), median (Q1, Q3)	17 (14.5, 24.5)	19 (13, 27)	0.747
Aspartate transaminase (U/L), mean ± SD	20.2 ± 7.3	29.3 ± 67.5	0.142
Activated partial thromboplastin time (s), mean ± SD	35 ± 3.9	35.4 ± 7.8	0.683
International normalized ratio, mean ± SD	1.1 ± 0.2	1 ± 0.2	0.293
Heart rate (bpm), mean ± SD	74.3 ± 14.9	70.9 ± 11.4	0.344
Systolic blood pressure (mmHg), mean ± SD	151.9 ± 20.3	142.1 ± 18.3	0.06
Diastolic blood pressure (mmHg), mean ± SD	83.1 ± 12.2	81.5 ± 10.5	0.592

### 3.2 Learning curve analysis

**Figure 1:**
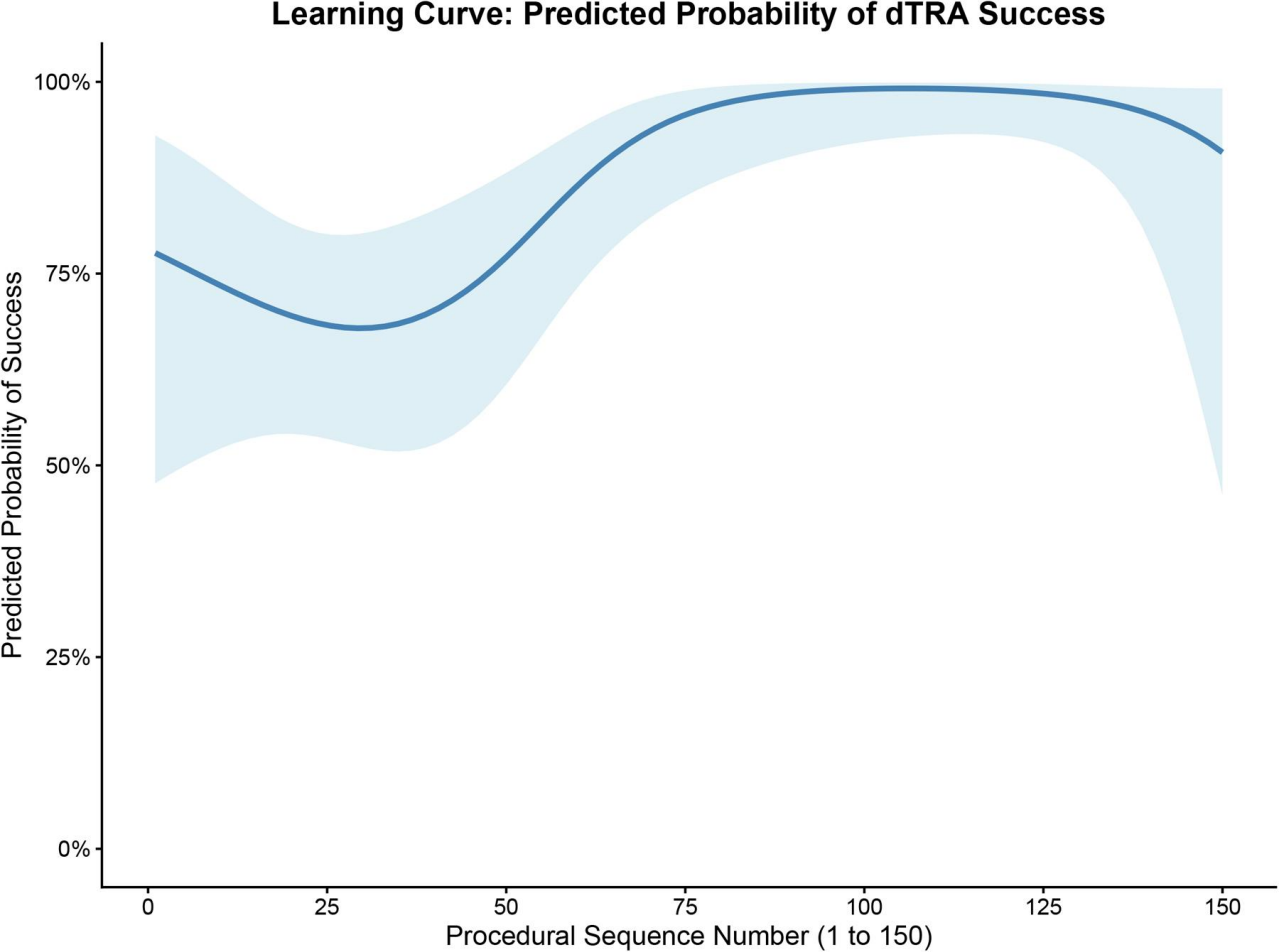
Learning curve for the probability of distal transradial access (dTRA) success.

**Figure 2:**
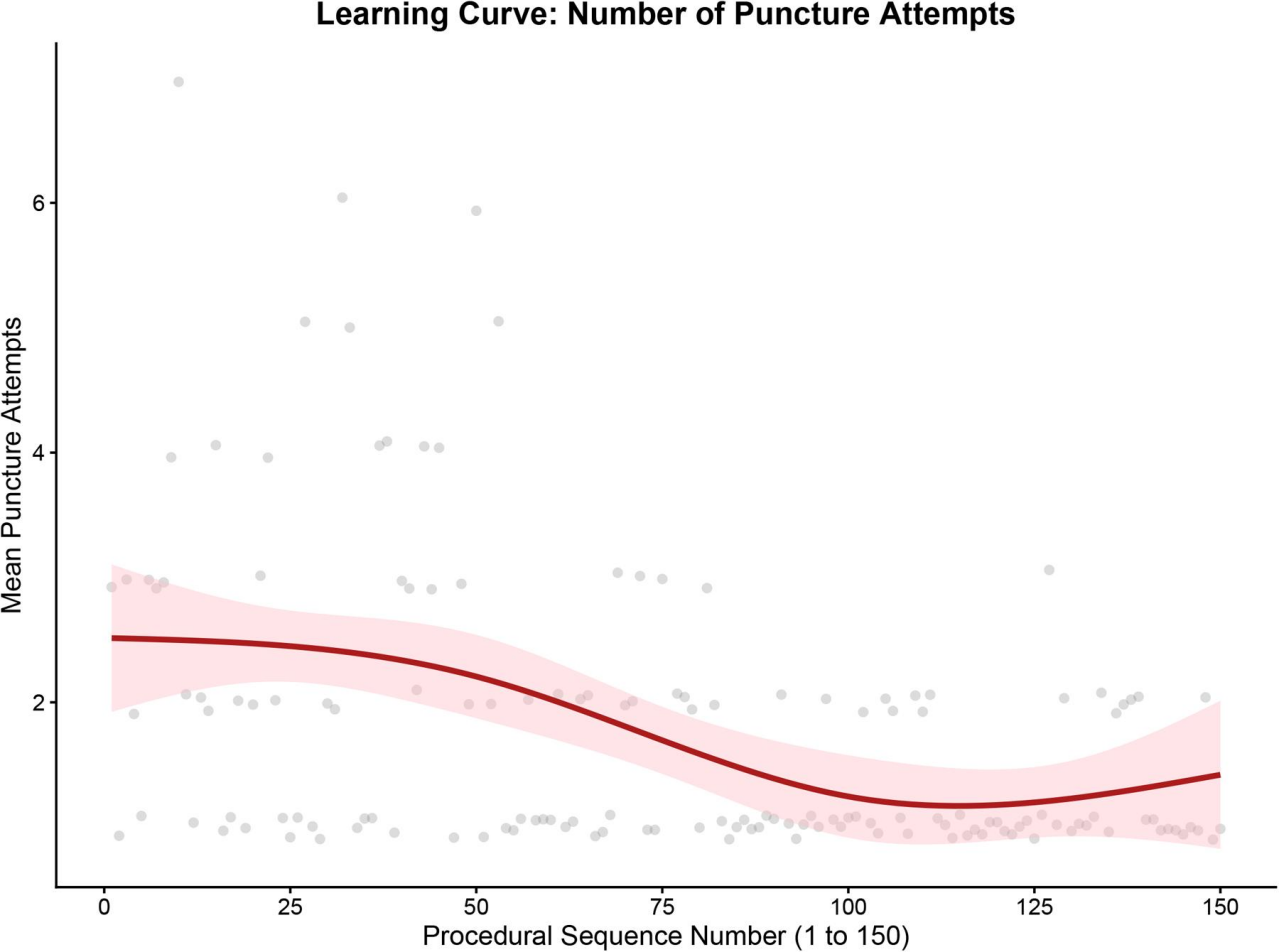
Learning curve for the number of puncture attempts.

**Figure 3:**
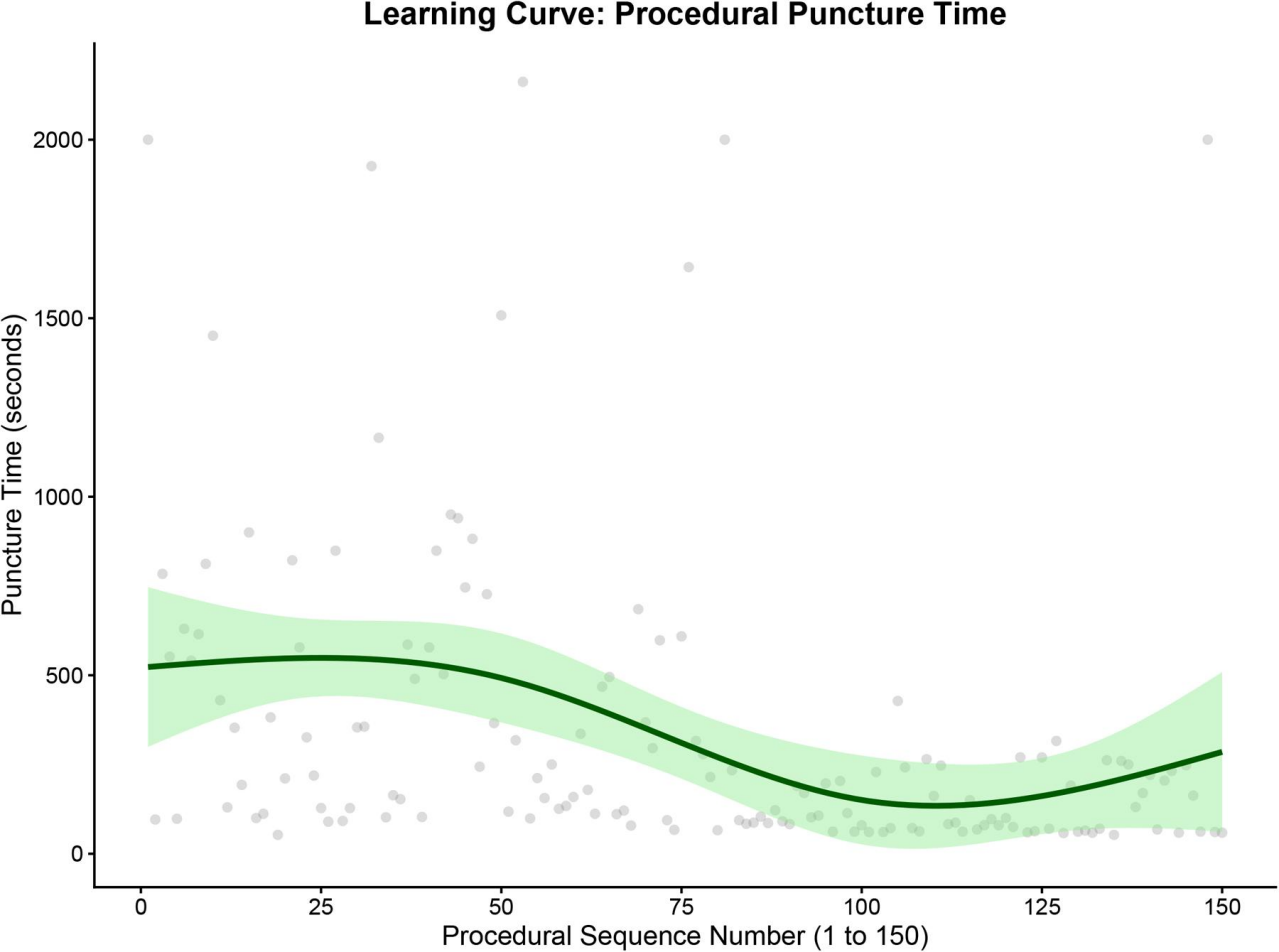
Learning curve for the procedural puncture time. The solid lines represent the restricted cubic spline fits, and the shaded areas indicate the 95% confidence intervals (CI). Scatter points in Figures 2, 3 represent raw data. dTRA, distal transradial access; CI, confidence interval.

To objectively evaluate the technical maturation of the operator, we utilized restricted cubic spline modeling to map the learning curve continuously across the 150 consecutive cases. The analysis revealed a clear two-phase learning effect. First, regarding procedural success, the predicted probability demonstrated a steep upward trajectory during the initial phase and plateaued after approximately 75 cases (Figure 1). Second, regarding procedural efficiency, the continuous spline models revealed a more prolonged optimization period. Both the mean number of puncture attempts (Figure 2) and the procedural puncture time (Figure 3) exhibited a gradual, continuous decline that stabilized later, reaching a plateau at approximately 100 cases. These results indicate that while the operator achieved fundamental proficiency and a stable success rate after 75 cases, maximum procedural efficiency and speed required approximately 100 cases.

### 3.3 Complications

No major complications requiring clinical intervention were observed in the 131 successful dTRA cases. Based solely on clinical assessment (e.g., pulse palpation), there were no instances of symptomatic radial artery occlusion during hospitalization or the clinical follow-up period (up to 12 months); however, routine post-procedural ultrasound was not performed. The most common complication was a minor local hematoma at the puncture site (*n* = 8, 5.3%), which were small subcutaneous bruises (EASY, Classes I and II) that resolved with compression (15). Only three patients (2%) reported transient puncture-related local numbness or pain, which resolved spontaneously before discharge. The EASY classification refers to the bleeding taxonomy set down in the ‘Early Discharge After Transradial Stenting of Coronary Arteries’ guidelines.

### 3.4 Analysis of the factors associated with dTRA failure

#### 3.4.1 Univariate analysis

The univariate logistic regression analysis results are summarized in Table 2. The analysis revealed that several factors were significantly associated with dTRA success. High levels of brain natriuretic peptide (BNP), admission SBP, and serum creatinine, as well as a history of coronary artery calcification and cerebral infarction, were negative predictors of puncture success (all *P* < 0.05). Additionally, a history of peripheral artery disease was associated with puncture failure (*P* = 0.057).

**Table 2:** Univariate logistic regression analysis for the predictors of dTRA puncture success (variables with *P* < 0.10).

Variable	Odds ratio (OR)	95% CI	*P*-value
Brain natriuretic peptide (pg/mL)	0.999	0.998–1.000	0.022
Systolic blood pressure (mmHg)	0.973	0.947–0.998	0.037
Coronary artery calcification	0.646	0.408–0.981	0.048
History of cerebral infarction	0.308	0.099–1.074	0.049
Serum creatinine (μmol/L)	0.984	0.967–1.000	0.049
Peripheral artery disease	0.348	0.120–1.095	0.057

CI, confidence interval; dTRA, distal transradial access.

#### 3.4.2 LASSO regression analysis

Following the univariate analysis, LASSO regression was performed to adjust for potential confounders, and the most critical predictors of dTRA failure were selected from numerous variables. During the 10-fold cross-validation, under the conservative *λ*. 1se standard, the model identified no predictors due to excessive penalization. However, using the *λ*. min standard, the analysis successfully revealed three variables significantly associated with outcomes (Table 3 and Supplementary Figure S1). The results indicate that the anterior-wall puncture technique was an independent protective technical factor, whereas high BNP and admission SBP levels were independent patient-related risk factors. The discriminative ability of this final LASSO model was satisfactory, yielding a C-statistic (AUC) of 0.794 (95% CI: 0.699–0.889) (Supplementary Figure S2).

**Table 3:** Factors associated with dTRA puncture success identified via LASSO regression analysis.

Selected variable	Coefficient (*β*)	Odds ratio (OR)
Puncture technique (Anterior wall)	0.881	2.41
Brain natriuretic peptide (per 100 pg/mL)	−0.0194	0.98
Systolic blood pressure (per 10 mmHg)	−0.00835	0.99

The intercept of the model is 1.767. OR was calculated as exp(*β*). For continuous variables, the coefficient and OR were scaled for clinical interpretation.

dTRA, distal transradial access; LASSO, least absolute shrinkage and selection operator; OR, odds ratio.

## 4 Discussion

This study yielded two primary findings. First, by employing continuous spline-based modeling, we quantitatively delineated a two-phase learning curve for the dTRA technique. For an experienced conventional TRA operator, fundamental proficiency—indicated by a stabilized, high probability of success—was achieved after approximately 75 cases. However, maximum procedural efficiency, characterized by the minimization of puncture attempts and time, required a more prolonged adaptation period, stabilizing at approximately 100 cases. This dissociation between achieving procedural success and optimizing procedural efficiency is a recognized phenomenon in surgical learning curves, reflecting the transition from ‘competence’ to ‘mastery’. Second, through LASSO regression analysis, we identified the anterior-wall puncture technique as a key protective factor and high baseline BNP and SBP levels as patient-related risk factors, significantly associated with access failure.

The learning curve milestone of approximately 75 cases observed in our study is notably shorter than the 200 cases reported by Roh et al. (11). This discrepancy may be attributed to the operator's extensive experience with conventional TRA (>2,000 cases), which possibly accelerated adaptation to the unique anatomical features and technical demands of the dTRA approach. Importantly, the observed improvement in the learning curve reflects not only increased manual dexterity but also a progressive evolution in puncture technique. The anterior-wall approach was not consistently applied from the beginning. Initially, using the conventional through-and-through technique, the operator frequently encountered scenarios where successful vessel puncture (indicated by blood return) was followed by failed guidewire advancement—likely due to the inherent tortuosity or spasm of the distal radial artery. This issue initially necessitated multiple puncture attempts (2–3 times) to achieve success. Consequently, the operator progressively adopted the anterior-wall puncture technique, which significantly improved the first-pass success rate of guidewire insertion. Therefore, this progressive technical transition undoubtedly drove the upward trajectory of the success rate and the continuous decline in total puncture attempts observed in our spline models.

The identification of high BNP and SBP levels as predictors of dTRA failure is a notable finding in our study. However, we must emphasize that the mechanistic link between these variables and puncture difficulty remains speculative. Elevated BNP may reflect the severity of underlying heart failure and suboptimal peripheral perfusion, but its direct association with distal radial artery size, vasoreactivity, or spasm is unclear. Similarly, while high SBP may act as a clinical surrogate for arterial stiffness—which could theoretically complicate vessel puncture by making the artery roll or resist the needle—arterial stiffness was not directly measured in our cohort. Therefore, elevated BNP and SBP should be strictly interpreted as clinical risk markers that signal a potentially challenging puncture scenario, rather than proven mechanistic drivers of failure. Interestingly, our finding that high SBP is a risk marker contradicts that of Roh et al., who identified low SBP (<120 mm Hg) as a risk factor (11). These contrasting results suggest a potential U-shaped relationship, where both very low blood pressure (resulting in a weak, poorly palpable pulse) and very high blood pressure (reflecting stiff, non-compliant vessels) may increase puncture difficulty. Furthermore, large-scale registry data have confirmed a strong correlation between operator experience and success rate (16).

The clinical and methodological implications of our study are two-fold. Regarding the clinical perspective, our findings provide a tangible target for dTRA training programs and underscore the importance of promoting the anterior-wall puncture techniques (17). Additionally, they suggest that operators should be particularly cautious when approaching patients with high baseline BNP or SBP levels. Regarding the methodological perspective, our study highlights the utility of LASSO regression in clinical scenarios with a few events. When traditional multivariate regression is limited by minimal events, LASSO may be introduced to effectively select the core predictors. This was particularly evident in our analysis. For example, univariate analysis revealed several potential predictors; however, only BNP and SBP were retained in the final LASSO model, suggesting that they are robust core predictors.

This study has some limitations. First, the single-center, single-operator design substantially limits external validity. The observed 75-case threshold primarily reflects the skill acquisition of a highly experienced conventional TRA operator (>5,000 cases) rather than a generalizable training standard. Operator-specific factors—such as advanced manual dexterity, high tactile sensitivity (critical without routine ultrasound guidance), and early adaptation to the anterior-wall technique—likely accelerated this learning process. Therefore, this threshold cannot be directly extrapolated to less experienced operators or trainees, who will likely require a longer learning curve. To enhance generalizability for beginners, we suggest the conventional through-and-through (double-wall) puncture technique combined with a soft PCI guidewire, which may be easier to adopt than the tactilely demanding anterior-wall approach. Furthermore, for low-volume centers or novice operators, we strongly recommend routine ultrasound guidance from the outset to flatten the learning curve. Second, due to the limited number of failure events (*n* = 19), we were statistically precluded from exploring complex interactions or potential non-linear relationships among baseline predictors, and residual confounding by factors such as vascular calcification cannot be entirely ruled out. Additionally, while we tracked puncture time and attempts to assess procedural difficulty, data regarding the specific use of ad-hoc adjunctive rescue maneuvers (such as localized vasodilator administration via the puncture needle) were not systematically recorded, which limits a fully comprehensive assessment of procedural challenges. Third, we relied on clinical evaluation (palpation up to 12 months and selected 12-month angiography) rather than systematic post-procedural ultrasound follow-up to detect vascular complications. As pulse palpation has limited sensitivity for detecting non-flow-limiting thrombi, this approach likely underestimates the true incidence of subclinical or asymptomatic RAO (18). Lastly, our study lacks pre-procedural ultrasound assessments of the distal radial artery diameter, which is widely recognized as a crucial determinant of dTRA success. Because pre-procedural ultrasound was not routinely performed during the study period, these anatomical data were unavailable and could not be retrospectively retrieved from imaging records. Future multicenter prospective studies should incorporate routine ultrasound assessments to validate our findings and enhance the accuracy of the predictive models.

## 5 Conclusion

An experienced operator in conventional transradial interventions may achieve proficiency in dTRA after approximately 75 procedures. The LASSO regression model revealed the anterior-wall puncture technique as a key procedural factor for improving success rates. At the patient level, high baseline BNP and SBP levels serve as clinical risk markers—rather than proven mechanistic drivers—that predict a higher probability of puncture failure.

## Data Availability

The raw data supporting the conclusions of this article will be made available by the authors, without undue reservation.
